# Biochemical Hallmarks of Oxidative Stress-Induced Overactivation of* Xenopus* Eggs

**DOI:** 10.1155/2019/7180540

**Published:** 2019-07-02

**Authors:** Alexander A. Tokmakov, Misaki Awamura, Ken-Ichi Sato

**Affiliations:** Department of Molecular Biosciences, Kyoto Sangyo University, Kamigamo-motoyama, Kita-ku, Kyoto 603-8555, Japan

## Abstract

Egg overactivation occurs with a low frequency in the populations of naturally ovulated frog eggs. At present, its natural inducers, molecular mechanisms, and intracellular events remain unknown. Using microscopic and biochemical analyses, we demonstrate here that high levels of hydrogen peroxide-induced oxidative stress can cause time- and dose-dependent overactivation of* Xenopus* eggs. Lipofuscin accumulation, decrease of soluble cytoplasmic protein content, and depletion of intracellular ATP were found to take place in the overactivated eggs. Progressive development of these processes suggests that egg overactivation unfolds in a sequential and ordered fashion.

## 1. Introduction

Oocytes and eggs of the African clawed frog* Xenopus laevis* provide the most common model for studying oogenesis, fertilization, meiotic and mitotic cell cycle progression, and apoptosis because of their large size and high biochemical tractability. The term “eggs” is generally used in the frog model for mature ovulated oocytes arrested in metaphase of the second meiotic division by high activity of the key meiotic regulators, such as the maturation promoting factor (MPF) and the cytostatic factor (CSF) [1]. The meiotic metaphase arrest prevents cell cycle progression and parthenogenesis prior to fertilization. Meiotically arrested eggs awaiting fertilization can experience various injuries leading to the loss of their quality. The stress- and age-triggered damage leads to decreased rates of fertilization, polyspermy, parthenogenesis, and abnormal development of embryos. Poor quality of oocytes and eggs is considered to be a cause of infertility and abnormal embryo development in different animals, including mammals [2, 3]. In addition, spontaneous egg activation and exit from the meiotic metaphase arrest make successful fertilization impossible [4, 5]. It was reported that unfertilized* Xenopus* eggs spontaneously activate, exit the meiotic arrest, and degrade by a robust apoptotic process within 48 hours after ovulation [6, 7].

The intracellular pathways involved in spontaneous egg activation are poorly investigated. It has been suggested that this process might engage a calcium-dependent mechanism in mammalian eggs [4, 8–10]. Indeed, artificial elevation of intracellular calcium concentration is known to initiate parthenogenetic activation of eggs in various species. However, spontaneous activation may also utilize calcium-independent mechanisms. In aging unfertilized sea urchin eggs, apoptosis was shown to be triggered by progressive inactivation of MAPK [11, 12]. Also, it was demonstrated that aged mouse and pig eggs have decreased activities of major CSF and MPF components [13, 14]. The gradual decrease in the content and/or activity of the key meiotic regulators below a threshold level necessary to maintain the meiotic metaphase arrest was hypothesized to cause meiotic exit in the absence of intracellular calcium signal [5].

At present, physiological inducers of spontaneous egg activation remain unidentified. It was suggested that oxidative stress might act as the initiator for a cascade of events that lead to expedited aging and deterioration of postovulatory oocytes [15]. Using* Xenopus* eggs, it was demonstrated previously that hydrogen peroxide initiates tyrosine phosphorylation and elevates intracellular calcium, resulting in Src kinase-dependent egg activation [16]. The study also reported that prolonged treatment with hydrogen peroxide led to excessive cortical contraction, egg swelling, and overactivation with a very distinctive egg phenotype. Studies of overactivated eggs are important because they can expand our understanding of cell death by unveiling alternative physiological mechanisms. So far, the intracellular events that occur in eggs upon overactivation have not been investigated in detail. Considering that hydrogen peroxide can easily diffuse through the cell plasma and subcellular compartment membranes to directly inflict oxidative damage [17], it could be expected that the drug might interfere with various intracellular processes and severely damage egg homeostasis.

In this work, we investigated oxidative stress-induced overactivation of* Xenopus* eggs using light and fluorescent microscopy, histochemical staining, protein, and bioluminescent assays. It was found that lipofuscin accumulation, decrease of soluble cytoplasmic protein content, and depletion of intracellular ATP occur in the eggs overactivated by strong oxidative stress. To the best of our knowledge, this is the first report concerning the changes of cellular homeostasis in overactivated eggs.

## 2. Materials and Methods

### 2.1. Materials

Anesthetic MS-222, water-soluble progesterone, and ATP Bioluminescence Assay Kit CLS II were purchased from Sigma (St. Louis, MO). Collagenase (280 U/mg) was obtained from Wako (Osaka, Japan) and human chorionic gonadotropin was from Teikoku Zoki (Tokyo Japan). Hydrogen peroxide, Sudan Black B (SBB), and protein assay CBB solution were from Nacalai Tesque (Kyoto, Japan). Hydrogen peroxide colorimetric/fluorometric assay kit was from BioVision (Milpitas, CA). Other chemicals were obtained from Wako and Nacalai Tesque. Slide glasses and cover slips for microscopy were purchased from Matsunami Glass (Osaka, Japan).

### 2.2. Animals, Cell, and Extracts

Adult female frogs of wild-type of* Xenopus laevis* were purchased from Shimizu (Kyoto, Japan) and maintained in dechlorinated water at the ambient temperature of 21-23°C. The experiments with the animals were conducted according to the Kyoto Sangyo University Animal Experimentation Regulations. Egg ovulation was induced by injection of 500 U/animal of human chorionic gonadotropin in the dorsal lymph sac of female frogs. Eggs were collected by squeezing the abdominal parts of the animals in about 10 hours after injection and kept in OR-2 buffer at the ambient temperature. Oocytes were isolated as described previously [6]. Briefly, frogs were anesthetized in 2 mg/ml solution of MS-222 and put on ice; then ovaries were surgically removed and placed into OR-2 solution containing 82.5 mM NaCl, 2.5 mM KCl, 1 mM CaCl_2_, 1 mM MgCl_2_, 1 mM Na_2_HPO_4_, 5 mM HEPES, and pH 7.6. Ovaries were manually dissected into clumps of 50–100 oocytes and extensively washed with OR-2 solution. Clumps of oocytes were treated with 5 mg/ml collagenase (280 U/mg) in OR-2 at 23°C for 2-3 hours by shaking at 60 rpm. Oocytes were extensively washed in OR-2 solution and left for stabilization over 4 h. Undamaged defolliculated oocytes of stage VI were manually selected and used in the experiments.* In vitro* oocyte maturation was induced by addition of 5 *μ*M progesterone and monitored by appearance of a white spot on the animal hemisphere of oocytes. To obtain cytosolic fractions, eggs were homogenized by pipetting in tenfold volume of cold OR-2 buffer containing protease inhibitors APMSF and leupeptin and then centrifuged at 10,000 rpm, 4°C, for 10 min. Supernatant fractions were collected and stored on ice until following biochemical analysis.

### 2.3. Microscopic Observations

Egg observation and imaging were carried out using SZX16 stereo zoom microscope (Olympus, Japan) equipped with high-frame digital microscope CCD camera DP73, CCD interface* U*-TV0.5XC-*3*, wide-angle objective SDF PLAPO 1xPF. The CellSens Standard software (Olympus) was used for image acquisition. Acquired images were further processed with the ImageJ software of the National Institute of Health [18] freely available at https://imagej.nih.gov/ij/.

### 2.4. Treatment of Eggs with Hydrogen Peroxide

Hydrogen peroxide was added at the specified concentrations (1-100 mM) to the oocytes matured* in vitro* for 10-12 hours in the presence of progesterone. The cells were washed with OR-2 buffer before peroxide administration to remove the hormone. The precise concentration of hydrogen peroxide was determined by titration using the hydrogen peroxide colorimetric/fluorometric assay kit, according to the manufacturer's manual.

### 2.5. Detection of Lipofuscin

Lipofuscin is a nondegradable aggregate of oxidized lipids and proteins that accumulates within lysosomes. Two major methods are currently employed for lipofuscin detection. One of them is based on evaluation of lipofuscin autofluorescence and another one on Sudan Black B (SBB) staining [19]. In this study, autofluorescence of lipofuscin in the insoluble particulate fraction of* Xenopus* eggs and staining of egg intracellular compartments with SBB were performed as described previously [20].

### 2.6. Measurements of Intracellular ATP

To measure intracellular ATP contents, the ATP Bioluminescence Assay Kit CLS II was used according to manufacturer's manual. Egg cytosolic fractions were obtained as described in Section 2.2. 1-*μ*l fraction aliquots were taken into 100-*μ*l bioluminescence assays. Intensity of luminescence was quantified using a GeneLight GL-220 compact luminometer (Microtec, Funabashi, Japan) within one minute after initiation of luciferase reaction by sample addition.

### 2.7. Other Methods

Protein content in egg cytosolic fractions was determined with the CBB protein assay. Sample absorbance was measured using a NanoDrop 1000 Spectrophotometer (Thermo Fisher, Waltham, MA). Bovine serum albumin was applied as a calibration standard. Quantified data in figures are presented as means ± SD values of four to six measurements taken in single-batch experiments. The experiments were repeated with the separate batches of oocytes and eggs obtained from four different animals. From 50 to 100 eggs were observed in the experiments that concerned counting overactivated egg phenotype.

## 3. Results

### 3.1. Overactivated Xenopus Eggs Are Distinguishable by Their Phenotype

**Figure 1:**
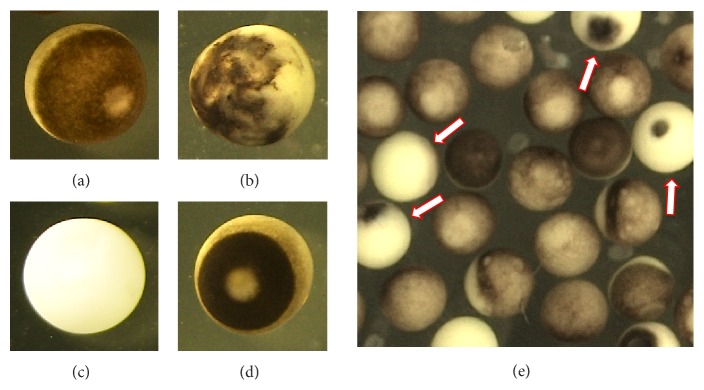
Morphological types of eggs observed in populations of naturally ovulated* Xenopus* eggs. Panels (a), (b), (c), and (d) present mature fertilization-competent, apoptotic, overactivated, and cortically contracted eggs, respectively. Panel (e) shows population of eggs matured* in vitro* in the presence of progesterone and aged by overnight incubation on bench. Arrows point to overactivated eggs.

Several types of eggs can be found in aging populations of ovulated unfertilized* Xenopus* eggs. The major types are presented in Figures 1(a)–1(d). They include the eggs arrested in the second meiotic metaphase, as it can be judged by the presence of a white spot on the dark animal hemisphere (Figure 1(a)); apoptotic eggs that lost the white spot after activation and experience progressive decoloring of the pigment layer (Figure 1(b)); overactivated eggs that lost their pigmentation and became near completely white (Figure 1(c)); and the eggs with the contracted pigment layer of the animal hemisphere (Figure 1(d)). The proportion of contracted eggs is usually low in egg populations because cortical contraction is transient and completes within 15 min (see next section for details). Importantly, overactivated eggs can clearly be distinguished by their specific phenotype in aging populations of frog eggs (Figure 1(e)), making easy their observation and collection for following biochemical analysis.

### 3.2. High Levels of Oxidative Stress Induce Time- and Dose-Dependent Overactivation of Xenopus Eggs

**Figure 2:**
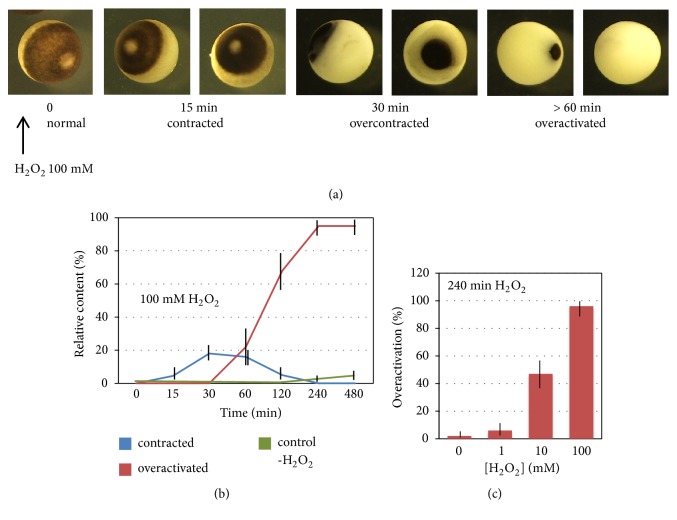
Time and dose dependencies of egg overactivation by hydrogen peroxide. Panel (a) shows dynamics of morphological changes in the eggs overactivated by 100 mM hydrogen peroxide. Panel (b) presents time course of egg cortical contraction and overactivation after addition of 100 mM hydrogen peroxide. The untreated control (-H_2_0_2_) in panel (b) refers to overactivated phenotype. Panel (c) shows dose dependency of egg overactivation. Overactivated phenotype in panel (c) was counted in 240 min after addition of peroxide.

The proportion of overactivated eggs in the populations of naturally ovulated frog eggs is quite low, not exceeding normally 2-3%. However, the mature meiotically arrested eggs can be effectively overactivated in the presence of millimolar concentrations of hydrogen peroxide. Strong oxidative stress induces fast cortical contraction of the egg pigment layer that can be detected within 15 min of peroxide administration (Figure 2(a)). In contrast to physiological egg activation, the cortical contraction induced by the prolonged incubation with hydrogen peroxide is not reversible. The pigmented area progressively shrinks, producing overcontracted phenotypes by 30 minutes. Further cortical contraction results in overactivated egg phenotypes with nearly complete loss of egg pigmentation (Figure 2(a)). The proportion of overactivated eggs increases steadily with time, approaching 100% within 4 hours of peroxide administration. Thus, oxidative stress induces time-dependent egg overactivation, and dynamics of this process is presented in Figure 2(b). In addition, the rate of egg overactivation also depends on concentration of hydrogen peroxide, as presented in Figure 2(c). In our experiments, the lowest effective concentration of peroxide capable of inducing overactivation was found to be 1 mM (Figure 2(c)).

### 3.3. Oxidative Stress Stimulates Lipofuscin Accumulation in Xenopus Eggs

The results presented in Figures 1 and 2 are based on observations of egg morphology. To gain a deeper insight into the oxidative stress-induced egg overactivation, we further attempted to pinpoint biochemical features of this process. As hydrogen peroxide is known to induce oxidation of proteins and lipids, the content of lipofuscin, a nondegradable aggregate of oxidized lipids, proteins, and metals, was investigated in the insoluble particulate fractions of peroxide-treated* Xenopus* eggs.

**Figure 3:**
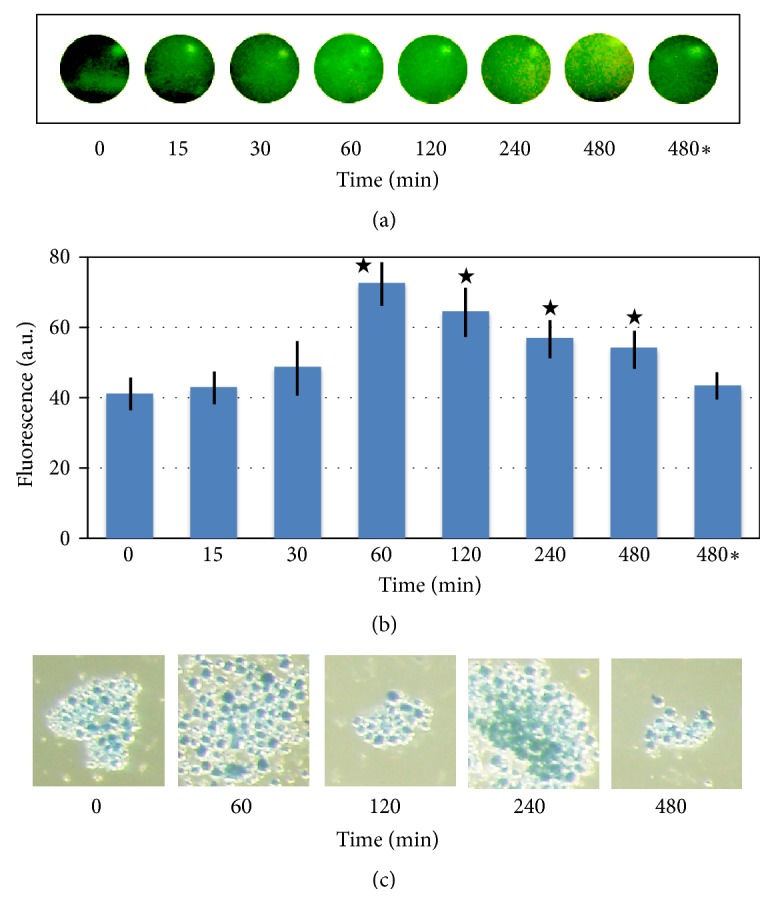
Oxidative stress-induced accumulation of lipofuscin in* Xenopus* eggs. Spot assay of lipofuscin autofluorescence in the insoluble particulate fractions of the peroxide-treated eggs and its quantification are presented in panels (a) and (b), respectively. The items labeled as 480*∗* refer to control eggs incubated for 480 min in the absence of peroxide. Stars in panel (b) indicate statistical significance from the untreated control (p<0.05). Panel (c) shows SBB staining of the egg endosomal compartment. Hydrogen peroxide (f.c. 100 mM) was added to the eggs at 0 time.

No significant changes in the lipofuscin level were observed in the eggs within 30 min of peroxide treatment; however lipofuscin content was elevated in the eggs incubated with peroxide for more than 1 hour, as revealed by monitoring lipofuscin-specific autofluorescence [Figures 3(a) and 3(b)]. Of note, a decrease in lipofuscin content was evident at the longer exposure times of 4 and 8 hours. This could probably be attributed to a gradual decrease of peroxide concentration in the egg incubation medium over that time. Indeed, it was found that peroxide concentration falls in the incubation dishes to about 30% of its original level after 8 hours (data not shown). The results of fluorescent analysis were further confirmed by an alternative method of lipofuscin detection. Staining egg particulate fractions with SBB, a lipophilic histochemical dye that reacts with lipids and lipofuscin, revealed color augmentation at 1 and 2 hours followed by color reduction at 4 and 8 hours of peroxide treatment [Figure 3(c)].

### 3.4. Strong Oxidative Stress Disrupts Protein and Energy Homeostases in Xenopus Eggs

**Figure 4:**
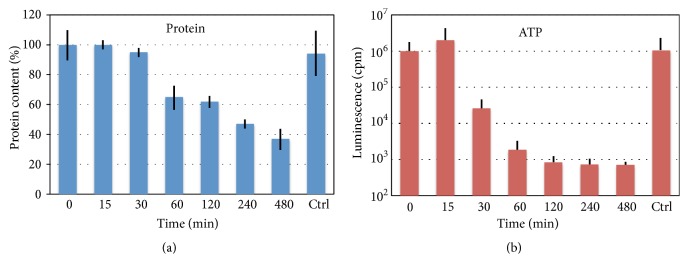
Protein and ATP contents in overactivated* Xenopus* eggs. The contents of soluble cytosolic protein and intracellular ATP in the eggs overactivated by addition of 100 mM hydrogen peroxide are shown in panels (a) and (b), respectively. Control (Ctrl) in the panels refers to the eggs incubated for 480 min in the absence of hydrogen peroxide.

At present, intracellular events that occur upon egg overactivation remain unknown. We hypothesized that the major metabolic traits, such as protein and energy homeostases, might be significantly affected by overactivation and measured the contents of soluble cytoplasmic protein and intracellular ATP in the hydrogen peroxide-treated eggs. A gradual decrease in the content of soluble protein and rapid drop in cellular ATP were observed in overactivated eggs (Figure 4). A statistically significant decrease in the protein content to about 60% level was registered in eggs after 1 hour of hydrogen peroxide treatment (Figure 4(a)), and nearly complete depletion of intracellular ATP (a decrease of almost two orders of magnitude) occurred within 30 minutes (Figure 4(b)). These findings indicate that the oxidative stress applied exerts highly detrimental impact on egg metabolism, resulting in progressive disruption of protein and energy homeostases.

## 4. Discussion

Egg overactivation happens with a low frequency in the populations of naturally ovulated* Xenopus* eggs (Figure 1). Also, quite rare, spontaneous overactivation of* Xenopus* eggs can be observed in the absence of activation stimuli* in vitro* (Figure 2(b)). Presently, it is viewed as a pathological, spontaneous, and uncontrollable process that renders eggs fertilization incompetent. The results of our study indicate that egg overactivation occurs in the time- and dose-dependent fashion in response to strong oxidative stress (Figure 2), resulting in progressive disruption of cellular homeostasis. Lipofuscin accumulation, protein degradation, and ATP depletion represent biochemical hallmarks of the oxidative stress-induced egg overactivation process (Figures 3 and 4).

Natural inducers of egg overactivation are unknown. In the present study, we used hydrogen peroxide to impose oxidative stress on eggs. It was found that high concentrations of peroxide (10-100 mM) elicit most robust and synchronized egg overactivation (Figure 2(c)), providing a convenient biochemically tractable model of strong oxidative stress. However, physiological relevance of these concentrations is questionable. It seems that much lower peroxide concentrations (below ~1 mM) are actually relevant. Indeed, the average intracellular steady-state concentration of peroxide was reported to be about 10 nM, and the blood plasma concentration was estimated to be 100–5000 times higher [21]. Thus, peroxide concentration can reach the submillimolar range in biological fluids, and it can contribute, hypothetically, to the low-frequency overactivation observed in naturally ovulated frog eggs. Of note, egg stability and sensitivity to oxidative stress vary significantly between experiments. For instance, sensitivity of the eggs to oxidative stress in a previous study [16] was substantially higher, with a threshold of 0.1 mM hydrogen peroxide vs 1 mM in the present work, reflecting, most probably, differences in egg quality between individual egg batches. It is well known that the quality of* Xenopus* eggs greatly depends on the health and husbandry conditions of the adult females producing the eggs. Factors that affect oocyte quality include season of the year, nutrition, lightning, and water quality [22, 23].

It is established that strong oxidative stress can damage lysosomes, mitochondria, and other intracellular compartments, directly and potently affecting cellular homeostasis. Reactive oxygen species were shown to induce chemical modification of lysosomal membrane lipids and proteins, as well as lysosomal membrane permeabilization [24]. Accordingly, our present study revealed accumulation of lipofuscin, a nondegradable aggregate of oxidized lipids, proteins, and metals, in the insoluble particulate fraction of hydrogen peroxide-treated eggs (Figure 3). It was reported recently that lipofuscin is predominantly localized in specialized large-sized late acidic endosomes that store protein and lipids in* Xenopus *eggs. These endosomes were identified in colocalization studies as a subpopulation of yolk platelets, the organelles abundantly present in frog oocytes and eggs [20].

In addition, oxidative damage is known to lead to disruption of mitochondrial function, loss of mitochondrial membrane potential, mitochondrial membrane permeabilization, inhibition of the respiratory chain and ATP production, and release of mitochondrial proteins to the cytoplasm [25]. Correspondingly, our study revealed the depletion of intracellular ATP in hydrogen peroxide-treated eggs within 30 minutes (Figure 4(b)), suggesting severe damage of mitochondrial function by oxidative stress at that time.

At present, executive mechanisms of egg overactivation and ensuing cell death are unknown. Although disruption of mitochondrial function and release of mitochondrial proteins, such as cytochrome C, can promote caspase-dependent apoptotic cell death, it is highly unlikely that apoptosis is involved in degradation of overactivated* Xenopus* eggs. The pace of egg degradation by an apoptotic process that unfolds in the eggs after their activation is much slower than that of the process initiated by overactivation. For example, ATP depletion in apoptotic eggs can be observed only after many hours following egg activation [6]; however it occurs within 30 minutes of oxidative stress-induced egg overactivation (Figure 3(b)). Of note, ATP depletion occurs typically quite late in apoptosis because high ATP levels are necessary to maintain this process [26].

Alternatively, oxidative stress has been reported to initiate autophagy, a homeostatic process that allows cells to degrade cytoplasmic proteins and organelles [27, 28]. For example, mitochondria damaged in Alzheimer disease by oxidative stress in neurons are subjected to autophagic degradation, leading to neurodegeneration [29]. Markedly, autophagy also requires certain levels of intracellular ATP, making this process incompatible with the early ATP depletion that takes place during egg overactivation (Figure 3(b)). In the future, it would be interesting to delineate intracellular molecular events in the overactivated eggs and elucidate the exact pattern of their cell death.

## 5. Conclusions

This study demonstrates that (i) overactivated* Xenopus* eggs have a distinctive phenotype in the populations of naturally ovulated unfertilized eggs, (ii) egg overactivation can be induced time- and dose-dependently by high levels of oxidative stress, and (iii) oxidative stress-induced overactivation is accompanied by progressive disruption of cellular protein and energy homeostases. It is revealed for the first time that lipofuscin accumulation, decrease of soluble cytoplasmic protein content, and depletion of intracellular ATP take place in the eggs overactivated by strong oxidative stress. These intracellular events may serve as biochemical markers of egg overactivation. In addition, it appears that egg overactivation unfolds as a sequential and ordered process. Further investigations are necessary to delineate in detail the sequence of intracellular events during this process.

## Data Availability

All the data are available from the corresponding author upon request.
